# Dynamic carbon 13 breath tests for the study of liver function and gastric emptying

**DOI:** 10.1093/gastro/gou068

**Published:** 2014-10-21

**Authors:** Leonilde Bonfrate, Ignazio Grattagliano, Giuseppe Palasciano, Piero Portincasa

**Affiliations:** ^1^Department of Biomedical Sciences and Human Oncology, Clinica Medica ‘A. Murri', University of Bari Medical School, Bari, Italy and ^2^Italian College of General Practitioners, Florence and Bari, Italy

**Keywords:** breath tests, stable isotope, hepatic metabolism, gastric motility, scintigraphy

## Abstract

In gastroenterological practice, breath tests (BTs) are diagnostic tools used for indirect, non-invasive assessment of several pathophysiological metabolic processes, by monitoring the appearance in breath of a metabolite of a specific substrate. Labelled substrates originally employed radioactive carbon 14 (^14^C) and, more recently, the stable carbon 13 isotope (^13^C) has been introduced to label specific substrates. The ingested ^13^C-substrate is metabolized, and exhaled ^13^CO_2_ is measured by mass spectrometry or infrared spectroscopy. Some ^13^C-BTs evaluate specific (microsomal, cytosolic, and mitochondrial) hepatic metabolic pathways and can be employed in liver diseases (i.e. simple liver steatosis, non-alcoholic steato-hepatitis, liver fibrosis, cirrhosis, hepatocellular carcinoma, drug and alcohol effects).

Another field of clinical application for ^13^C-BTs is the assessment of gastric emptying kinetics in response to liquids (^13^C-acetate) or solids (^13^C-octanoic acid in egg yolk or in a pre-packed muffin or the ^13^C-*Spirulina platensis* given with a meal or a biscuit). Studies have shown that ^13^C-BTs, used for gastric emptying studies, yield results that are comparable to scintigraphy and can be useful in detecting either delayed- (gastroparesis) or accelerated gastric emptying or changes of gastric kinetics due to pharmacological effects. Thus, ^13^C-BTs represent an indirect, cost-effective and easy method of evaluating dynamic liver function and gastric kinetics in health and disease, and several other potential applications are being studied.

## Introduction

Breath tests (BTs) are diagnostic tools based on the ingestion of various substrates that are processed at different levels in the gastrointestinal tract [1]. The principle of BTs relies on the concept that the metabolized substrate leads to the production of gases (e.g. CO_2_, H_2_) that pass into the blood, are excreted and quantified in expired air. The interest towards the use of BTs has increased since they are relatively simple, safe and non-invasive tools with potential applications in several clinical conditions including liver diseases, *H. Pylori* infection, gastrointestinal motility, small intestinal bacterial overgrowth, and sugar (fructose, lactose) malabsorption. BTs using specific substrates labelled with the stable (non-radioactive) isotope ^13^C have been also employed for the assessment of hepatic functional reserve and to measure gastric emptying. The potential applications of such ^13^C-BTs will be discussed in the present paper.

## ^13^C breath tests for the study of liver function

### General features

A major challenge in clinical hepatology is the accurate and non-invasive assessment of liver function in patients with chronic liver disease. The evaluation of liver status is currently based on serum parameters of synthesis (prothrombin, cholesterol, albumin), hepatocellular integrity (transaminases), detoxification (ammonium), excretion and cholestasis (bilirubin, alkaline phosphatase, γGT), associated with imaging techniques (ultrasonography, computerized tomography, magnetic resonance and elastography) or scores such as the Child-Turcotte-Pugh classification, which combines clinical (ascites and degree of encephalopathy) and serum (bilirubin, albumin, and prothrombin time) parameters [2]. Such ‘static' tests of liver function may have limitations, especially when dealing with prediction of outcomes and in assessing liver dysfunction in critically ill patients [3]. In this respect, BTs represent novel indirect ‘dynamic' tools that provide additional insights in functional diagnosis and follow-up of patients with liver diseases [4].

The principles of BTs in hepatology are based on both biochemical and pharmacological considerations. Mechanisms of liver damage often include dysfunction of subcellular organelles, such as microsomal hypertrophy, mitochondrial abnormalities, and activation of peroxisomal metabolism (i.e. long chain fatty acids). Thus, assessing specific functions of such organelles through BTs may provide useful information to clinicians. Also, BTs allow the study of specific time-dependent metabolic processes by assessing the hepatic clearance of metabolically active substances [5]. In this context, for a given exogenous substrate:

HEPATIC CLEARANCE=HEPATIC PERFUSION×HEPATIC EXTRACTION

(where HEPATIC EXTRACTION is the ratio of the difference between inflow and outflow concentration ÷ by inflow concentration of the probe) [6].

Hepatic clearance is defined as flow-limited (range 0.7–1.0) or enzyme-limited (<0.3) [7]. The general characteristics of the ‘ideal' substrate for the study of liver function are depicted in Table 1 [8].

**Table 1.** General characteristics required of an ideal substrate for studying dynamic liver function

Pharmacokinetic and metabolic aspects
Rapidly and consistently absorbed when administered orally
Primarily metabolized in the liver
Low (20–30%) hepatic extraction ratio (i.e. metabolism independent from liver blood flow)
Clear metabolic pathway; simple pharmacokinetic; short elimination half-life
^ 13^CO_2_ generated should be distributed in the body, not compartmentalized
Methodological aspects
Safe
Simple to prepare and administer
No- or minimal interaction with extra-hepatic tissues (i.e. adipose tissue or muscle)
Reproducible over time and repeatable (useful for follow-up)
Costs
Low-priced

**Figure 1.**
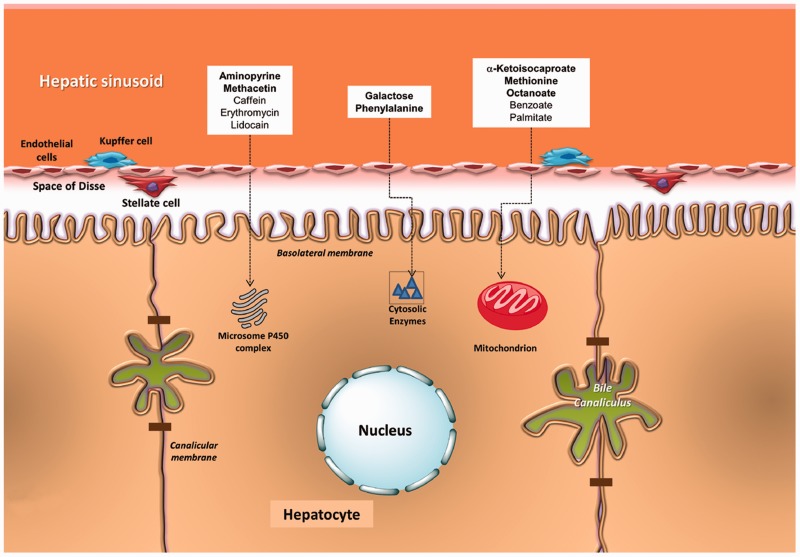
Sites where metabolic processes may be explored by breath test in hepatocytes. In particular, ^13^C-α-ketoisocaproic acid, ^13^C-methionine, and ^13^C-octanoate are the three substrates more widely employed for the dynamic assessment of mitochondrial function (see text for details).

**Figure 2.**
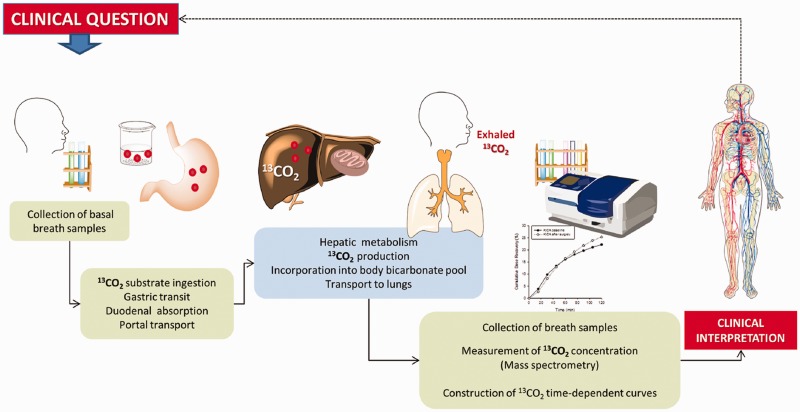
General methodology of breath test analysis using ^13^C-labelled substrates for the dynamic study of liver function and of gastric emptying, both depending on time-dependent concentration of exhaled ^13^CO2. The estimation of liver function is accurate if gastric emptying, duodenal absorption, portal transfer of the substrate to the liver, ^13^CO_2_ distribution in the body compartments, and lung function are preserved. The estimation of gastric emptying is accurate if liver function is also preserved.

The intrinsic complexity of liver metabolic pathways does not allow a single functional test to explore the whole liver function. Different substrates are therefore used to assess cytosolic, microsomal or mitochondrial function (Figure 1). Such substrates are marked with the natural stable isotope of carbon, carbon 13 (^13^C; currently the most widely used isotope). After intestinal absorption, the given substrate undergoes liver metabolism at different levels, which ultimately results in the production and appearance of ^13^CO_2_ in exhaled air, as a marker of specific liver metabolic functions [4, 9] (Figure 2).

### Breath tests for the study of liver cytosolic function

Phenylalanine is an aromatic amino acid, converted to tyrosine in the cytosol of the hepatocytes by the enzyme phenylalanine hydroxylase. Phenylalanine oxidation is affected when liver function is impaired and by using a ^13^C-phenylalanine BT, a reduced ^13^CO_2_ release in the exhaled breath is recorded [10, 11]. The ^13^C-phenylalanine BT has been used to predict post-operative complications and to monitor liver regeneration after partial hepatectomy, and to predict the severity of liver cirrhosis, staged according to the Child-Turcotte-Pugh score [12]. However, in a study by our group that compared the hepatic functional mass in chronic liver disease classified according to Child-Turcotte-Pugh score and serum bile acid levels, the diagnostic power of ^13^C-phenylalanine BT was less than that of ^13^C-methacetin BT [13].

Galactose is a carbohydrate primarily metabolized by the liver. The metabolism of galactose investigates the activity of galactose kinase, which catalyses the ATP-dependent phosphorylation of galactose to galactose 1-phosphate. Originating from the radioactive carbon 14 (^14^C)-galactose BT, the non-radioactive ^13^C-galactose BT has been also developed [14]. The performance of the ^13^C-galactose BT relates inversely with the severity of liver disease—and in particular with cirrhosis—reaching 71% sensitivity, 85% specificity, and 84% accuracy [15, 16]. Combination with another BT investigating the microsomal function (aminopyrine) further increases the diagnostic sensitivity and specificity [15]. The ^13^C-Galactose BT has also been used to assess progressive decline in liver mass function in patients with chronic liver disease due to Hepatitis C virus (HCV) infection [17].

### Breath tests for the study of liver microsomal function

Aminopyrine is known as an antipyretic analgesic drug. The ^13^C-labelled methyl groups of aminopyrin are demethylated with transformation into formate, formaldehyde, and the produced bicarbonate releases ^13^CO_2_ which is exhaled in breath. After oral administration, aminopyrine is completely absorbed and has a low hepatic extraction (E = 0.2), seen to be independent of liver blood flow and not disturbed by hepatic vascular shunts. Aminopyrine is metabolized by the hepatic microsomal cytochromes and provides an index of functional hepatic mass. Aminopyrine BTs have been used to provide prognostic information, to study, graft rejection, severity of paracetamol intoxication and to detect alcoholic liver injury. When used to discriminate the degree of liver fibrosis, aminopyrine BT displayed good specificity and sensitivity for advanced fibrosis or cirrhosis but performed poorly for intermediate fibrosis stages [7, 18, 19]. Some limitations need to be considered in the interpretation of aminopyrine BT: (i) the age of the subject can affect the P450-dependent N-demethylation, (ii) the simultaneous use of drugs with enzyme inductive effect or inhibiting the microsomal enzymatic activity might alter the findings of the BT, and (iii) female sex hormones can produce a negative effect on aminopyrine metabolism [6, 20].

Methacetin is a derivative of phenacetin which is metabolized rapidly by the hepatic microsomal enzyme systems CYP1A2 into acetaminophen and ^13^CO_2_ by a single O-dealkylation step. Since methacetin has a high extraction (E > 0.8) [21] and undergoes extensive first-pass clearance, its metabolism can be altered by hepatic blood flow alterations and by hepatic 'first-pass' effect. Capacity for metabolizing methacetin is lower in elderly people than in other adults [22]. Methacetin BT was shown to accurately assess the degree of liver damage in patients with histologically proven chronic liver diseases and to distinguish chronic aggressive hepatitis from liver cirrhosis; it also distinguished early cirrhosis (Child A) from non-cirrhotic patients. Methacetin BT was a useful predictive marker of clinical outcomes in chronic HCV patients [23–25]. Moreover, methacetin BT can better estimate the degree of fibrosis in patients with chronic HCV infection than biochemical parameters (i.e. aspartate aminotransferase-to-platelet ratio and aspartate aminotransferase-to-alanine aminotransferase ratio) or Fibroindex [26, 27].

### Breath tests for the study of liver mitochondrial function

**Figure 3.**
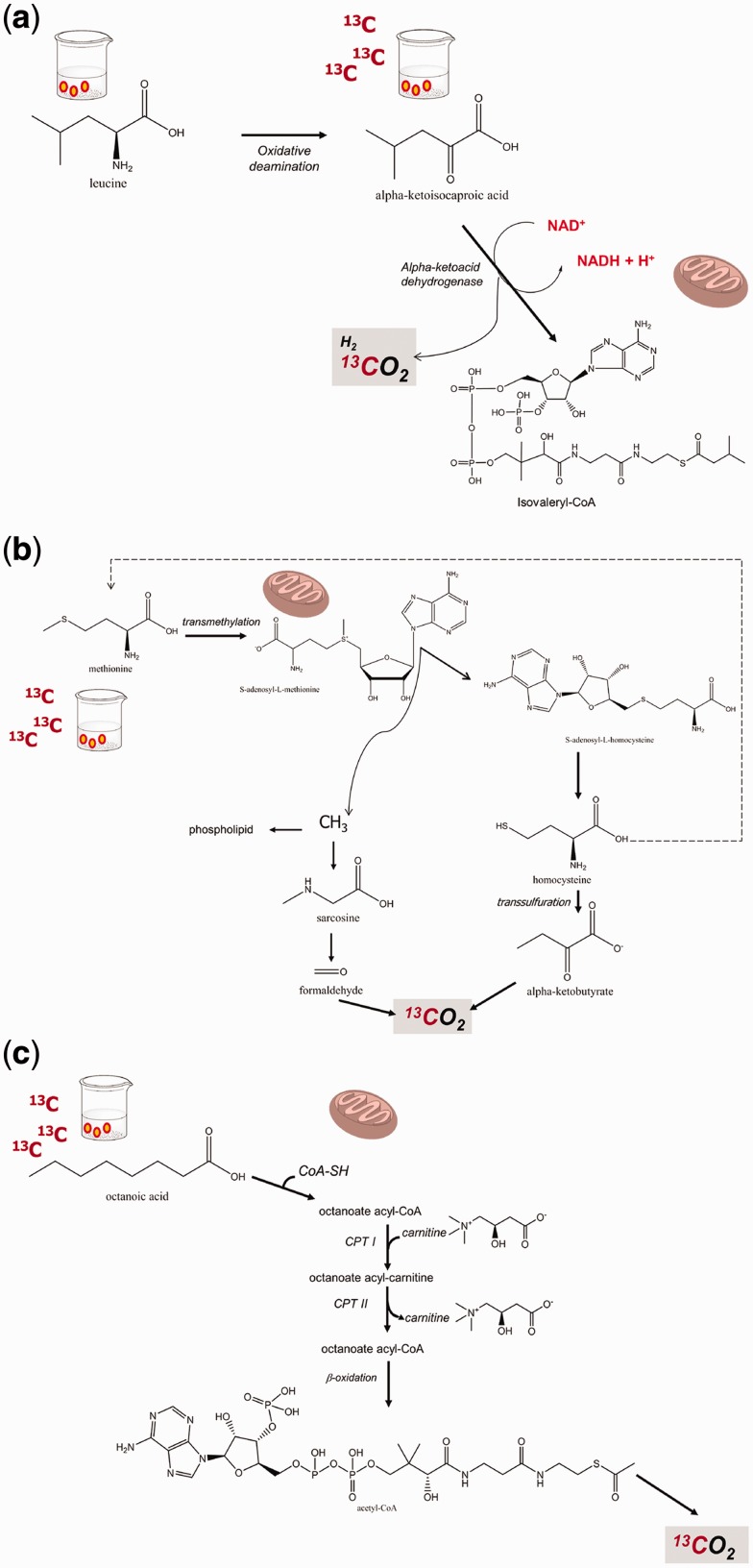
Mitochondrial metabolism of a) methionine; b) α-ketoisocaproic acid; c) octanoic acid. CO_2_ is invariably produced at the end of the process. The use of ^13^C-labelled substrates ultimately leads to production of ^13^CO_2_ following mitochondrial metabolism.

Ketoisocaproate (KICA) is an intermediate in the metabolism of leucine. The decarboxylation of KICA and the generation of CO_2_ reflects the mitochondrial branched-chain amino acid decarboxylation function [28]. This step is observed when transamination to leucine (the major competing pathway for KICA elimination) is suppressed by the simultaneous administration of fixed doses of leucine (Figure 3a). This metabolic pathway of KICA has been tested in experimental models, in isolated mitochondria, in healthy subjects treated with acetyl salicylic acid or with low ethanol intake, and in patients with liver diseases [8, 9, 28, 29]. ^13^C-KICA decarboxylation is lower in alcoholic patients than in patients with non-alcoholic fatty liver disease (NAFLD) or controls [29, 30]. We found that the mitochondrial decarboxylation capacity of KICA was lower in patients with advanced non-alcoholic steatohepatitis (NASH) than in healthy subjects and patients with simple liver steatosis. Notably, the ^13^CO_2_ cumulative recovery values following ^13^C-KICA were inversely related to the extent of fibrosis, to serum hyaluronate, and to body size in NASH patients [31]. We extended the studies with ^13^C-KICA BTs and found that KICA decarboxylation was significantly lower in cirrhotic patients with hepatocellular carcinoma (HCC) than in cirrhotic patients without HCC and identical Child-Pugh score. Moreover, KICA decarboxylation was deranged following radiofrequency ablation, but not after transarterial chemoembolization. Finally, the recurrence of HCC was associated with an early decrease of KICA decarboxylation [32]. In a different context, we recently found that a ^13^C-KICA BT was abnormal (and therefore suggesting mitochondrial malfunction) in a female patient suffering from massive liver echinococcosis. Notably, mitochondrial liver function improved following pericystectomy and limited hepatectomy [33]. KICA BT is useful for the assessment of drug effects on liver mitochondrial function. Liver injury might occur following the use of such drugs, which accumulate in the mitochondria and interfere with respiratory complexes or electron transfer [34, 35]. KICA BT may be helpful in ascertaining the integrity of these organelles before the administration of potentially toxic drugs and in detecting drug-induced mitochondrial damage before the appearance of symptoms, in order to manage patients in a timely manner and prevent adverse effects. Examples are tacrolimus, aspirin, and ergot alkaloids. There are also potential applications with amiodarone, valproate, and retroviral drugs [36, 37].

Methionine is an essential amino acid, principally metabolized in the liver with production of CO_2_ [38]. ^13^C-methionine, either in the form of L-(1-^13^C) methionine or (methyl-^13^C)-methionine, investigates the oxidative capacity of the liver [39] (Figure 3b). In human studies, ^13^C-methionine BT has been used to assess mitochondrial function during acute intoxication and in patients with chronic liver diseases. For example, acute ethanol consumption impairs ^13^C-methionine decarboxylation in healthy volunteers [39], whereas metabolism of methionine is decreased in patients with liver cirrhosis, and especially in those with ethanol aetiology, in patients with NAFLD, in those taking high-dose valproate or nucleoside analogues for the treatment of HIV, and in patients with Friedreich ataxia [37, 40–44]. A defective methionine metabolism has also been reported in hepatitis C-infected cells [45].

Octanoate is a medium chain fatty acid that enters mitochondria independently of the carnitine transport system. Within the mitochondria, octanoate undergoes β-oxidation, which generates acetyl co-enzyme A (AcCoA). AcCoA enters the Krebs cycle and is oxidized to CO_2_ unless utilized for the synthesis of other energy-rich compounds (Figure 3c). The ^13^C-octanoate BT has been employed for the study of gastric emptying (see below) but the test should also reflect hepatic mitochondrial function when gastric emptying and duodenal absorption are not severely deranged. Octanoate is therefore a potential substrate for non-invasive BT of hepatic mitochondrial β-oxidation. In fact, in animal models, the decarboxylation of octanoate was decreased in rats developing thioacetamide-induced acute hepatitis and liver cirrhosis [46]. In patients with NASH, the oxidation of octanoate was unchanged or increased [47, 48], and unchanged in those with early stage and advanced cirrhosis with and without porto-systemic shunt [49].

## ^13^C breath tests for the study of gastric emptying

### General features

A subgroup of patients may develop disturbed gastric motility disorders, namely accelerated ('dumping syndrome') or delayed gastric emptying, which is the most frequently investigated condition. Gastroparesis implies a delayed gastric emptying without mechanical obstruction and is a condition frequently associated with symptoms such as nausea, vomiting, bloating, early satiety, and or upper abdominal pain [50]. After excluding mechanical obstruction by means of upper endoscopy, computed tomography, barium follow-through examination or magnetic resonance enterography, the diagnosis of gastroparesis is based on functional studies to assess gastric motility in response to liquids and/or solid test meals. Investigations include the traditional scintigraphic studies [50], functional ultrasonography [51, 52], magnetic resonance imaging and, recently, wireless motility capsule [53] and BTs (see below). Major limitations of such techniques consist of radiation hazards (scintigraphy), duration of the exam and experience of the operator (ultrasonography), and costs (magnetic resonance imaging and wireless capsule). To establish the aetiology of gastroparesis a further step should take into account the idiopatic form (affecting about half of the patients), diabetes mellitus, and post-surgical, viral, neurological, autoimmune, viral causes, scleroderma, as well as effect of medications (e.g. calcium channel blockers, dopamine antagonists, octreotide, etc.). To establish the aetiology, laboratory tests, gastroduodenal manometry, autonomic testing and additional studies—including single photon emission computed tomography and full thickness biopsies of the stomach and small intestine—might be necessary from case to case.

### Breath tests for the study of gastric emptying

Gastric emptying has also been investigated by BTs using the radioactive isotope ^14^C and more recently, the non-radioactive stable isotope ^13^C, both labelling substrates dissolved in liquids (acetate in water or fruit juice) or solid test meals (octanoic acid in egg yolk). Octanoic acid, a medium-chain fatty acid fully retained in egg yolk during mixing and grinding in the stomach, is rapidly liberated in the duodenum and quickly transported to the liver via the *vena portae*. The ^13^C-octanoic acid rapidly enters the mitochondria without requiring carnitine (see also above: liver BTs) and is rapidly oxidized and metabolized to ^13^CO_2_. The assumption of the ^13^C-BT is that small intestinal absorption of the substrate, liver metabolism, and pulmonary excretion of ^13^CO_2_ must be preserved and rapid. Thus, a delay in the appearance of ^13^CO_2_ in breath samples is exclusively due to a reduced solid transit pace between the stomach and the duodenum, i.e. gastric emptying becomes the rate-limiting step when the substrate is rapidly absorbed. An important diagnostic parameter is the half-emptying time, i.e. the time at which half of the solid meal has moved from the stomach to the duodenum [54–59]. Breath samples are taken at baseline and every 30 min over a period of 240 min, using a non-linear regression formula [55] or, less frequently (e.g. at 45, 150 and 180 mins) during a period of 180 min using the generalized linear regression model [60] of the ^13^CO_2_ excretion-time curve. Measurement of exhaled ^13^CO_2_ is performed by mass spectrometry or infrared spectroscopy. Smoking, physical exercise, drinking and eating are prohibited during the test, and gastric motility-altering drugs are not allowed. The retention of ^13^C-octanoic acid in the solid meal should be confirmed by *in vitro* incubation studies [57, 61].

Several researchers have employed the *C-BTs (where * denotes the variable isotope number) for the study of gastric emptying, as summarized in Table 2. Results vary according to the number of healthy subjects and type of patients enrolled, methodology and type of *C isotope, test meal characteristics (liquid, solid, calorie content or preparation), and interpretation of results. Two mathematical models—the non-linear regression model [55] and the general linear regression model [62]—have been proposed for the calculation of the BT half-emptying time, starting from the time-dependent ^13^CO_2_ excretion curve. Several studies have compared BT with scintigraphy and a few have performed studies investigating the absorption kinetics of *C-octanoic after duodenal instillation or the retention of octanoic in specific meals.

**Table 2.** Principal studies investigating gastric emptying of liquids and solids by breath tests using carbon ^14^C and ^13^C

Author (year)	Subjects/patients	Isotope	Test meal (kcal)	Main diagnostic outcomes
Ghoos *et al.* (1993) [55]	16 healthy subjects 20 functional dyspepsia	^14^C-OA in egg yolk	Solid (320 kcal)	Validation studies of OA incorporation into egg yolk Validation with scintigraphy Non-linear regression model Subtraction of 66 min from ‘real' BT T1/2 calculated by mathematical model^a^ Tlag and GEC (Normal GEC = 3.1)
	5 healthy individuals	^14^C-OA and ^13^C-OA in egg yolk	Solid (320 kcal)	Repeated studies (3 times) Inter-individual variability NS Day-to-day variability NS Coefficient variation mean 27%
	41 healthy individuals	^a^C-OA in egg yolk	Solid (320 kcal)	Studies for normal range 72 ± 22SD min 83 min (75^th^ percentile)
Maes *et al.* (1994) [73, 74]	9 healthy subjects: accelerated emptying (i.v. 200 mg erythromycin); delayed emptying (i.v. 30 mg propantheline) 36 healthy subjects 20 dyspeptic patients	^14^C-OA and ^13^C-OA in egg yolk	Solid (250 kcal)	Validation with scintigraphy Non-linear regression model T1/2 Normal 71 ± 27SD min Accelerated 37 ± 21 min Delayed 141 ± 88 min
Braden *et al.* (1995) [64]	20 healthy subjects 16 functional dyspepsia	^13^C-acetate in 100 mL water	Liquid (0 kcal)	Validation with scintigraphy Normal T1/2: 78 ± 14 min Dyspepsia T1/2: 100 ± 21 min
Duan *et al.* (1995) [63]	6 healthy subjects 6 dyspeptic patients With or without cisapride (10 mg x 2) 12 healthy subjects (day-to-day variability)	^13^C-acetate in water ^13^C-OA in egg yolk	Liquid (0 kcal) Solid (250 kcal)	Liquid T1/2 83 ± 6SD min (control); 96 ± 21 min (dyspepsia) Solid T1/2 148 ± 35 min (control); 203 ± 41 min (dyspeptic) Cisapride T1/2 117 ± 27 min (control); 166 ± 58 min (dyspeptic) In healthy controls, T1/2 for liquids and solids were reproducible on the two different days
Choi *et al.* (1997) [62]	15 healthy subjects	^13^C-OA in egg yolk	Solid (240 kcal)	Validation with scintigraphy Non-linear linear regression model High intra-individual variability, dependent also on collection periods (4, 5, 6 hours, delta 43-63 min) Low intra-individual variability (on 3 different days) T1/2 median (range) 186–191 min (167–210)
Choi *et al.* (1998) [76]	30 healthy subjects	^13^C-OA in egg yolk	Solid (240 kcal)	Validation with scintigraphy T1/2: different between subjects but highly reproducible within subjects Normal T1/2 median 191 min (range 120–386)
Lee *et al.* (2000) [60]	30 healthy subjects	^13^C-*S. Platensis*	Solid (220 kcal) or Pre-packed biscuit	Validation with scintigraphy General linear regression model Normal T1/2 100 ± 20SD min (solid meal) T1/2 91 ± 15 min (biscuit)
Lee *et al.* (2000) [67]	6 healthy subjects 50 healthy subjects (additional) 22 symptomatic diabetic patients	^13^C-OA in egg yolk	Solid (420 kcal)	Validation with scintigraphy Non-linear regression model: high variability and T1/2 BT longer than T1/2 scintigraphy General linear regression models: more accurate results Normal T1/2 median 118 min (range 72–188) Diabetic gastroparesis in 3 patients (1 misclassified according to scintigraphy)
Viramontes *et al.* (2001) [68]	50 healthy subjects Accelerated emptying (i.v. erythromycin) Delayed emptying (i.v. atropine)	^13^C-*S. Platensis*	Solid (220 kcal)	Validation with scintigraphy General linear regression model T1/2 ‘Normal' range 70–150 min
Hellmig *et al.* (2006) [65]	90 healthy subjects	^13^C-acetate in water ^13^C-OA in egg yolk	Liquid (250 mL apple juice) Solid (310 kcal)	Liquid T1/2 81 ± 22SD min (range 43–51) Solid T1/2 144 ± 55 (median 127 min; 25–75% percentiles: 112.0–168 min) No influence of age, sex or BMI
Szarka *et al.* (2008) [69]	38 healthy subjects 5 healthy subjects (i.v. atropine) 124 patients (suspicion of delayed gastric emptying)	^13^C-*S. Platensis*	Solid (238 kcal)	Validation with scintigraphy Normal T1/2 68 ± 15SD Accelerated if <10^th^ percentile (52 min) Delayed if >90^th^ percentile (86 min)
Perri *et al.* (2010) [57]	131 healthy subjects 8 diabetic gastroparesis 11 untreated celiac patients	^13^C-OA	Solid (378 kcal) (EXPIROGer®) pre-packed muffin	T1/2 Normal 88 ± 29SD min Normal upper cut-off value 146 min Gastroparesis 179 ± 50 min Celiac disease 151 ± 20 min

^a^Probably dependent on the observed half-time for absorption and oxidation of OA after intraduodenal instillation (i.e. 62 min).

BMI = body mass index; BT = breath test; GEC = gastric emptying coefficient; NS = not significant; OA = octanoic acid; SD = standard deviation; T1/2 = half-emptying; Tlag = calculation of lag phase time (min).

A 150 mg dose of ^13^C-acetate has been used in the liquid test [63–65]. In solid test meals, different formulations have been employed:

1. One egg yolk labelled with 91–100 mg ^13^C-octanoic acid, served with 50 g of ham, 10 g of butter, two slices of white bread and a glass of water [equalling 324 kcal with 26 g carbohydrates (32%), 16 g fats (44%), and 19 g proteins (24%)] [54–56, 66] or slightly different calorie composition ranging from 220 kcal [60] to 420 kcal [67].
2. An edible blue-green alga protein-enriched food supplement (*Spirulina platensis*) labelled with 100–200 mg ^13^C-*spirulina*. The supplement is incorporated into egg white and the meal consists of either a cooked egg served with skimmed milk and wheat bread (220 kcal, protein 35%, carbohydrate 40%, fat 25% and 2.6 g of fibre) or as biscuit meal with a rye roll (160 kcal), cream cheese (90 kcal) and white grape juice (80 kcal) [60, 68, 69].
3. A new gluten-, glucose-, and lactose-free muffin (EXPIROGer®, Sofar, Milano, Italy) pre-labelled with 100 mg ^13^C-octanoic acid (equalling 378 kcal with 57 g carbohydrate (61%), 14 g fat (33%), and 6 g protein (6%)) [57].
4. Three small gluten-, glucose-, and lactose-free pre-packed muffins [equalling 390 kcal with 47 g carbohydrate (48%), 20 g fat (46%), and 5.5 g protein (6%)] ingested two before and one after a soluble capsule containing 75 mg ^13^C-octanoic acid (AB Analitica SrL, Padua, Italy) [70].

Table 2 also shows that BTs have been tested in patients with functional 'dysmotility-like' non-ulcer dyspepsia, idiopatic or diabetic gastroparesis, celiac disease, and accelerated or delayed emptying. Other studies have dealt with patients suffering from connective tissue disorders, gastro-oesophageal reflux disease, post-gastric surgery, dumping syndrome, HIV infection, gastric ulcer or obesity [55, 57, 64, 69]. The effects of drugs (cisapride, erythromycin atropine, propantheline and capsaicin) on gastric kinetics have been also investigated [63, 65, 68, 71–73].

The results depicted in Table 2 show a wide range of subjects/patients ranging in number from 6 to more than 130 and different outcomes in both health and disease. For example, for a liquid test meal, ^13^C-acetate yielded a mean half-emptying time of 78 ± 14 min in a study with 20 healthy subjects (coefficient of variation 10–36%) and showed a highly reproducible positive linear correlation with technetium-99 (^99m^Tc)-albumin colloid scintigraphy [64]. Data were confirmed in subsequent studies [63, 65].

In patients with functional dyspepsia, the mean half-emptying time was significantly delayed (Table 2) and showed a greater inter-individual variability [63, 64]. The authors concluded that the ^13^C-acetate BT is a reliable, widely applicable diagnostic tool to assess the gastric emptying of liquids and liquid phases in semi-solid test meals.

Starting from a seminal study in healthy subjects and dyspeptic patients, Ghoos *et al.* used ^14^C- and ^13^C-OA in egg yolk to validate the BTs according to the non-linear regression model, providing three kinetic parameters: half-emptying time, lag phase and gastric emptying coefficient [55]. Inter-individual and day-to-day variabilities were acceptable. The normal range of gastric emptying in healthy subjects was 72 ± 22SD min. Further studies have used BTs during pharmacologically-induced accelerated or delayed gastric emptying [72, 73].

When validating the ^13^C-*Spirulina platensis* solid test meal in 30 healthy volunteers, the Mayo Clinic group found optimal correlation *vs.* scintigraphy, even when restricting the measurements to a few time points (i.e. baseline, 75, 90 and 180 min, i.e. the Mayo 'reduced model') [60]. The half-emptying time was 100 ± 20SD min the solid meal and 91 ± 15 min with the *S. platensis* biscuit. In a subsequent study in 57 healthy subjects, simulated disturbances of gastric emptying were induced by either accelerating (using i.v. erythromycin) or delaying (using i.v. atropin) gastric emptying. As compared with scintigraphy and normal half-emptying values (95 ± 24 min) the sensitivity and specificity of ^13^C-*Spirulina platensis* BT using the solid meal were 86% and 80%, respectively, in detecting either accelerated (71 ± 14 min) or delayed emptying (207 ± 44 min), using a normal range for half-emptying time of 70–150 min [68].

In the multicentre Italian study employing the pre-packed solid meal EXPIROGer®, we recently found that the reference range of half-emptying time in 131 healthy subjects was 88 ± 29SD min with a normal upper cut-off value of 146 min. There was no significant difference between subjects sorted by sex or age. The within-subject variability of half-emptying time was 17%. By contrast, the half-emptying values were significantly delayed, showing 179 ± 50 min in diabetic patients with gastroparesis and 151 ± 20 min in untreated celiac disease patients [57]. In the initial series of healthy subjects using the novel three-muffins test, the ^13^C-octanoic BT yielded a half-emptying time of 108 ± 18SD (Portincasa *et al.*: unpublished observations) [70].

Using the ^13^C stable isotope represents a simple, non-invasive, office- or field-based technique [60, 74]. The accuracy of BTs for the study of gastric emptying appears to be comparable with scintigraphic studies [60, 64, 69, 73], but the technique, although easy to perform, fully non-invasive, and extendable also to children and pregnant women, represents an indirect method of assessing gastric emptying. In other words, variability in the rate of absorption, metabolism, and excretion of the marker between individuals must be considered in the interpretation of data. Imprecision with both stable isotope and scintigraphy in measuring gastric emptying, however, does reflect pathophysiological variations. The use of BTs for the study of gastric emptying appears promising for intra-individual comparisons. Further studies, however, might further improve standardization of the methodology in terms of statistical analysis, time-test, and sampling frequency [57, 60, 66, 75, 76].

## Conclusions and future perspectives

BTs represent valuable diagnostic non-invasive tools for *in vivo* assessment of various enzyme activities, transport processes, or organ functions. By monitoring the metabolization of several ^13^C-labelled substrates and the consequent appearance of ^13^CO_2_ in breath, it is possible to study the liver function dynamically (focussing on different intracellular pathways) as well as gastric emptying kinetics (similarly to scintigraphy, regarded as the ‘gold standard'). BTs currently use the non-radioactive stable isotope carbon ^13^C as tracer, which is safe in children, during pregnancy and while breast-feeding. BTs are simple, non-radioactive office- or field-based tools which, in epidemiological and pharmachodynamic studies, can better define pathophysiologically relevant abnormalities in the fields of hepatology and gastroenterology. Interpretation of BTs requires a knowledge of methodological limitations and potential pitfalls before routinely extending such studies in the clinical setting.

*Conflict of interest statement*: none declared.

## References

[1] Gasbarrini A Corazza GR Gasbarrini G Methodology and indications of H2-breath testing in gastrointestinal diseases: the Rome Consensus Conference. Aliment Pharmacol Ther 2009;29 Suppl 1:1–49. 19344474 10.1111/j.1365-2036.2009.03951.x

[2] Sherlock S Dooley J Diseases of the liver and biliary system. Oxford: Blackwell Science, 2002:597–628.

[3] Sakka SG Assessing liver function. Curr Opin Crit Care 2007;13:207–14. 17327744 10.1097/MCC.0b013e328012b268

[4] Grattagliano I Lauterburg BH Palasciano G 13C-breath tests for clinical investigation of liver mitochondrial function. Eur J Clin Invest 2010;40:843–50. 20597965 10.1111/j.1365-2362.2010.02331.x

[5] Merkel C Bolognesi M Bellon S Aminopyrine breath test in the prognostic evaluation of patients with cirrhosis. Gut 1992;33:836–42. 1624169 10.1136/gut.33.6.836 PMC1379346

[6] Miele L Marrone G Cefalo C Potential use of liver function breath tests in the clinical practice. Eur Rev Med Pharmacol Sci 2013;17 Suppl 2:82–9. 24443073

[7] Giannini E Fasoli A Chiarbonello B 13C-aminopyrine breath test to evaluate severity of disease in patients with chronic hepatitis C virus infection. Aliment Pharmacol Ther 2002;16:717–25. 11929389 10.1046/j.1365-2036.2002.01200.x

[8] Armuzzi A Candelli M Zocco MA Review article: breath testing for human liver function assessment. Aliment Pharmacol Ther 2002;16:1977–96. 12452932 10.1046/j.1365-2036.2002.01374.x

[9] Michaletz PA Cap L Alpert E Assessment of mitochondrial function in vivo with a breath test utilizing alpha–ketoisocaproic acid. Hepatology 1989;10:829–32. 2807162 10.1002/hep.1840100513

[10] Burke PA Stack JA Wagner D L-[1-(13)C] Phenylalanine oxidation as a measure of hepatocyte functional capacity in end–stage liver disease. Am J Surg 1997;173:270–4. 9136778 10.1016/S0002-9610(96)00392-3

[11] Lara Baruque S Razquin M Jimenez I 13C-phenylalanine and 13C-methacetin breath test to evaluate functional capacity of hepatocyte in chronic liver disease. Dig Liver Dis 2000;32:226–32. 10975773 10.1016/s1590-8658(00)80825-7

[12] Zhang GS Bao ZJ Zou J Clinical research on liver reserve function by 13C-phenylalanine breath test in aged patients with chronic liver diseases. BMC Geriatr 2010;10:23. 20459849 10.1186/1471-2318-10-23 PMC2875214

[13] Festi D Capodicasa S Sandri L Measurement of hepatic functional mass by means of 13C-methacetin and 13C-phenylalanine breath tests in chronic liver disease: comparison with Child-Pugh score and serum bile acid levels. World J Gastroenterol 2005;11:142–8. 15609414 10.3748/wjg.v11.i1.142 PMC4205374

[14] Shreeve WW Shoop JD Ott DG Test for alcoholic cirrhosis by conversion of [14C]- or [13C]galactose to expired CO2. Gastroenterology 1976;71:98–101. 1278655

[15] Giannini EG Fasoli A Borro P 13C-galactose breath test and 13C-aminopyrine breath test for the study of liver function in chronic liver disease. Clin Gastroenterol Hepatol 2005;3:279–85. 15765448 10.1016/s1542-3565(04)00720-7

[16] Saadeh S Behrens PW Parsi MA The utility of the 13C-galactose breath test as a measure of liver function. Aliment Pharmacol Ther 2003;18:995–1002. 14616165 10.1046/j.1365-2036.2003.01753.x

[17] Giannini E Botta F Borro P Relationship between thrombopoietin serum levels and liver function in patients with chronic liver disease related to hepatitis C virus infection. Am J Gastroenterol 2003;98:2516–20. 14638357 10.1111/j.1572-0241.2003.08665.x

[18] Rocco A de Nucci G Valente G 13C-aminopyrine breath test accurately predicts long–term outcome of chronic hepatitis C. J Hepatol 2012;56:782–7. 22173159 10.1016/j.jhep.2011.10.015

[19] Schneider AR Teuber G Paul K Patient age is a strong independent predictor of 13C-aminopyrine breath test results: a comparative study with histology, duplex-Doppler and a laboratory index in patients with chronic hepatitis C virus infection. Clin Exp Pharmacol Physiol 2006;33:300–4. 16620291 10.1111/j.1440-1681.2006.04365.x

[20] Opekun AR Jr. Klein PD Graham DY [13C]Aminopyrine breath test detects altered liver metabolism caused by low-dose oral contraceptives. Dig Dis Sci 1995;40:2417–22. 7587824 10.1007/BF02063247

[21] Brockmoller J Roots I Assessment of liver metabolic function. Clinical implications. Clin Pharmacokinet 1994;27:216–48. 7988103 10.2165/00003088-199427030-00005

[22] Ciccocioppo R Candelli M Di Francesco D Study of liver function in healthy elderly subjects using the 13C-methacetin breath test. Aliment Pharmacol Ther 2003;17:271–7. 12534413 10.1046/j.1365-2036.2003.01413.x

[23] Klatt S Taut C Mayer D Evaluation of the 13C-methacetin breath test for quantitative liver function testing. Z Gastroenterol 1997;35:609–14. 9297776

[24] Liu YX Huang LY Wu CR Measurement of liver function for patients with cirrhosis by 13C-methacetin breath test compared with Child-Pugh score and routine liver function tests. Chin Med J (Engl) 2006;119:1563–6. 16996011

[25] Zipprich A Meiss F Steudel N 13C-Methacetin metabolism in patients with cirrhosis: relation to disease severity, haemoglobin content and oxygen supply. Aliment Pharmacol Ther 2003;17:1559–62. 12823160 10.1046/j.1365-2036.2003.01604.x

[26] Koda M Matunaga Y Kawakami M FibroIndex, a practical index for predicting significant fibrosis in patients with chronic hepatitis C. Hepatology 2007;45:297–306. 17256741 10.1002/hep.21520

[27] Dinesen L Caspary WF Chapman RW 13C-methacetin-breath test compared to also noninvasive biochemical blood tests in predicting hepatic fibrosis and cirrhosis in chronic hepatitis C. Dig Liver Dis 2008;40:743–8. 18339592 10.1016/j.dld.2008.01.013

[28] Parra D Gonzalez A Mugueta C Laboratory approach to mitochondrial diseases. J Physiol Biochem 2001;57:267–84. 11800289 10.1007/BF03179820

[29] Lauterburg BH Liang D Schwarzenbach FA Mitochondrial dysfunction in alcoholic patients as assessed by breath analysis. Hepatology 1993;17:418–22. 8444415

[30] Witschi A Mossi S Meyer B Mitochondrial function reflected by the decarboxylation of [13C]ketoisocaproate is impaired in alcoholics. Alcohol Clin Exp Res 1994;18:951–5. 7978109 10.1111/j.1530-0277.1994.tb00065.x

[31] Portincasa P Grattagliano I Lauterburg BH Liver breath tests non-invasively predict higher stages of non-alcoholic steatohepatitis. Clin Sci (Lond) 2006;111:135–43. 16603025 10.1042/CS20050346

[32] Palmieri VO Grattagliano I Minerva F Liver function as assessed by breath tests in patients with hepatocellular carcinoma. J.Surg.Res. 2009;157:199–207. 19540521 10.1016/j.jss.2008.09.029

[33] Bonfrate L Giuliante F Palasciano G Unexpected discovery of massive liver echinococcosis. A clinical, morphological, and functional diagnosis. Ann Hepatol 2013;12:634–41. 23813143

[34] Pessayre D Mansouri A Haouzi D Hepatotoxicity due to mitochondrial dysfunction. Cell Biol Toxicol 1999;15:367–73. 10811531 10.1023/a:1007649815992

[35] Pessayre D Fromenty B Berson A Central role of mitochondria in drug–induced liver injury. Drug Metab Rev 2012;44:34–87. 21892896 10.3109/03602532.2011.604086

[36] Gabe SM Bjarnason I Tolou-Ghamari Z The effect of tacrolimus (FK506) on intestinal barrier function and cellular energy production in humans. Gastroenterology 1998;115:67–74. 9649460 10.1016/s0016-5085(98)70366-x

[37] Sternfeld T Lorenz A Schmid M Increased red cell corpuscular volume and hepatic mitochondrial function in NRTI-treated HIV infected patients. Curr HIV Res 2009;7:336–9. 19442131 10.2174/157016209788347985

[38] Finkelstein JD Methionine metabolism in mammals. J Nutr Biochem 1990;1:228–37. 15539209 10.1016/0955-2863(90)90070-2

[39] Armuzzi A Marcoccia S Zocco MA Non-invasive assessment of human hepatic mitochondrial function through the 13C-methionine breath test. Scand J Gastroenterol 2000;35:650–3. 10912667 10.1080/003655200750023633

[40] Spahr L Negro F Leandro G Impaired hepatic mitochondrial oxidation using the 13C–methionine breath test in patients with macrovesicular steatosis and patients with cirrhosis. Med Sci Monit 2003;9:CR6–11. 12552242

[41] Milazzo L Piazza M Sangaletti O [13C]Methionine breath test: a novel method to detect antiretroviral drug–related mitochondrial toxicity. J Antimicrob Chemother 2005;55:84–9. 15590719 10.1093/jac/dkh497

[42] Sternfeld T Lorenz A Schmid M [(13)C]Methionine breath test as a marker for hepatic mitochondrial function in HIV-infected patients. AIDS Res Hum Retroviruses 2009;25:1243–8. 20001311 10.1089/aid.2009.0013

[43] Banasch M Ellrichmann M Tannapfel A The non-invasive (13)C-methionine breath test detects hepatic mitochondrial dysfunction as a marker of disease activity in non–alcoholic steatohepatitis. Eur J Med Res 2011;16:258–64. 21810560 10.1186/2047-783X-16-6-258 PMC3353401

[44] Stuwe SH Goetze O Arning L Hepatic mitochondrial dysfunction in Friedreich ataxia. BMC Neurol 2011;11:145. 22085827 10.1186/1471-2377-11-145 PMC3226643

[45] Feld JJ Modi AA El–Diwany R S-adenosyl methionine improves early viral responses and interferon-stimulated gene induction in hepatitis C nonresponders. Gastroenterology 2011;140:830–9. 20854821 10.1053/j.gastro.2010.09.010 PMC3021477

[46] Shalev T Aeed H Sorin V Evaluation of the 13C-octanoate breath test as a surrogate marker of liver damage in animal models. Dig Dis Sci 2010;55:1589–98. 19731033 10.1007/s10620-009-0913-2

[47] Schneider AR Kraut C Lindenthal B Total body metabolism of 13C-octanoic acid is preserved in patients with non-alcoholic steatohepatitis, but differs between women and men. Eur J Gastroenterol Hepatol 2005;17:1181–4. 16215429 10.1097/00042737-200511000-00005

[48] Miele L Grieco A Armuzzi A Hepatic mitochondrial beta-oxidation in patients with nonalcoholic steatohepatitis assessed by 13C-octanoate breath test. Am J Gastroenterol 2003;98:2335–6. 14572600 10.1111/j.1572-0241.2003.07725.x

[49] van de Casteele M Luypaerts A Geypens B Oxidative breakdown of octanoic acid is maintained in patients with cirrhosis despite advanced disease. Neurogastroenterol Motil 2003;15:113–20. 12680910 10.1046/j.1365-2982.2003.00397.x

[50] Camilleri M Parkman HP Shafi MA Clinical guideline: management of gastroparesis. Am J Gastroenterol 2013;108:18–37. 23147521 10.1038/ajg.2012.373 PMC3722580

[51] Portincasa P Moschetta A Baldassarre G Pan-enteric dysmotility, impaired quality of life and alexithymia in a large group of patients meeting the Rome II criteria for irritable bowel. World J Gastroenterology 2003;9:2293–9. 10.3748/wjg.v9.i10.2293 PMC4656481 14562396

[52] Di Ciaula A Wang DQ Portincasa P Gallbladder and gastric motility in obese newborns, pre-adolescents and adults. J Gastroenterol Hepatol 2012;27:1298–305. 22497555 10.1111/j.1440-1746.2012.07149.x

[53] Kuo B McCallum RW Koch KL Comparison of gastric emptying of a nondigestible capsule to a radio-labelled meal in healthy and gastroparetic subjects. Aliment Pharmacol Ther 2008;27:186–96. 17973643 10.1111/j.1365-2036.2007.03564.x

[54] Ghoos Y Geypens B Rutgeerts P 13CO2 breath tests and stable isotopes for investigating gastrointestinal functions. Acta Gastroenterol.Belg. 2000;63:325–7. 11233513

[55] Ghoos YF Maes BD Geypens BJ Measurement of gastric emptying rate of solids by means of a carbon-labeled octanoic acid breath test. Gastroenterology 1993;104:1640–7. 8500721 10.1016/0016-5085(93)90640-x

[56] Maes BD Geypens BJ Ghoos YF 13C-Octanoic acid breath test for gastric emptying rate of solids. Gastroenterology 1998;114:856–9. 9547107 10.1016/s0016-5085(98)70608-0

[57] Perri F Bellini M Portincasa P (13)C-octanoic acid breath test (OBT) with a new test meal (EXPIROGer): Toward standardization for testing gastric emptying of solids. Dig Liver Dis 2010;42:549–53. 20116352 10.1016/j.dld.2010.01.001

[58] Perri F Clemente R Festa V 13C-octanoic acid breath test: a reliable tool for measuring gastric emptying. Ital J Gastroenterol Hepatol 1998;30:211–17. 9675662

[59] Perri F Ghoos YF Maes BD Gastric emptying and Helicobacter pylori infection in duodenal ulcer disease. Digestive Diseases and Sciences 1996;41:462–8. 8617116 10.1007/BF02282319

[60] Lee JS Camilleri M Zinsmeister A A valid, accurate, office based, non–radioactive test for gastric emptying of solids. Gut 2000;46:768–73. 10807886 10.1136/gut.46.6.768 PMC1756454

[61] Bromer MQ Kantor SB Wagner DA Simultaneous measurement of gastric emptying with a simple muffin meal using [13C]octanoate breath test and scintigraphy in normal subjects and patients with dyspeptic symptoms. Dig Dis Sci 2002;47:1657–63. 12141833 10.1023/a:1015856211261

[62] Choi MG Camilleri M Burton DD [13C]octanoic acid breath test for gastric emptying of solids: accuracy, reproducibility, and comparison with scintigraphy. Gastroenterology 1997;112:1155–62. 9097998 10.1016/s0016-5085(97)70126-4

[63] Duan LP Braden B Caspary WF Influence of cisapride on gastric emptying of solids and liquids monitored by 13C breath tests. Dig Dis Sci 1995;40:2200–6. 7587790 10.1007/BF02209007

[64] Braden B Adams S Duan LP The [13C]acetate breath test accurately reflects gastric emptying of liquids in both liquid and semisolid test meals. Gastroenterology 1995;108:1048–55. 7698571 10.1016/0016-5085(95)90202-3

[65] Hellmig S Von Schöning F Gadow C Gastric emptying time of fluids and solids in healthy subjects determined by13C breath tests: influence of age, sex and body mass index. J Gastroenterol Hepatol 2006;21:1832–8. 17074022 10.1111/j.1440-1746.2006.04449.x

[66] Delbende B Perri F Couturier O 13C-octanoic acid breath test for gastric emptying measurement. Eur J Gastroenterol Hepatol 2000;12:85–91. 10656216 10.1097/00042737-200012010-00016

[67] Lee JS Camilleri M Zinsmeister AR Toward office-based measurement of gastric emptying in symptomatic diabetics using [13C]octanoic acid breath test. Am J Gastroenterol 2000;95:2751–61. 11051344 10.1111/j.1572-0241.2000.03183.x

[68] Viramontes BE Kim DY Camilleri M Validation of a stable isotope gastric emptying test for normal, accelerated or delayed gastric emptying. Neurogastroenterol Motil 2001;13:567–74. 11903917 10.1046/j.1365-2982.2001.00288.x

[69] Szarka LA Camilleri M Vella A A stable isotope breath test with a standard meal for abnormal gastric emptying of solids in the clinic and in research. Clin Gastroenterol Hepatol 2008;6:635–43 e1. 18406670 10.1016/j.cgh.2008.01.009 PMC3739971

[70] Bonfrate L Ruggiero V Mossel E A novel promising solid test meal to study gastrointestinal kinetics and symptoms in health and disease. A pilot study by functional ultrasonography. European Journal of Clinical Investigation 2014;44:78.

[71] Debreceni A Abdel-Salam OME Figler M Capsaicin increases gastric emptying rate in healthy human subjects measured by 13C-labeled octanoic acid breath test. J Physiol Paris 1999;93:455–60. 10674924 10.1016/s0928-4257(99)00114-x

[72] Maes BD Hiele MI Geypens BJ Pharmacological modulation of gastric emptying rate of solids as measured by the carbon labelled octanoic acid breath test: influence of erythromycin and propantheline. Gut 1994;35:333–7. 8150342 10.1136/gut.35.3.333 PMC1374585

[73] Maes BD Ghoos YF Rutgeerts PJ [*C]octanoic acid breath test to measure gastric emptying rate of solids. Dig Dis Sci 1994;39(12 Suppl):104S–6S. 7995200 10.1007/BF02300385

[74] Portincasa P Palasciano G Breath testing for gastric emptying and intestinal transit evaluation. Gastroenterol Int 1999;12:46–8.

[75] Shin AS, Camilleri M. Diagnostic assessment of diabetic gastroparesis. Diabetes 2013;62:2667–73. 23881199 10.2337/db12-1706 PMC3717858

[76] Choi MG Camilleri M Burton DD Reproducibility and simplification of 13C-octanoic acid breath test for gastric emptying of solids. Am J Gastroenterol 1998;93:92–8. 9448183 10.1111/j.1572-0241.1998.092_c.x

